# Transcriptomic Changes Related to Cellular Processes with Particular Emphasis on Cell Activation in Lysosomal Storage Diseases from the Group of Mucopolysaccharidoses

**DOI:** 10.3390/ijms21093194

**Published:** 2020-04-30

**Authors:** Estera Rintz, Lidia Gaffke, Magdalena Podlacha, Joanna Brokowska, Zuzanna Cyske, Grzegorz Węgrzyn, Karolina Pierzynowska

**Affiliations:** Department of Molecular Biology, University of Gdansk, Wita Stwosza 59, 80-308 Gdansk, Poland; estera.rintz@gmail.com (E.R.); lidia.gaffke@phdstud.ug.edu.pl (L.G.); magdalena.podlacha@biol.ug.edu.pl (M.P.); joanna.brokowska@phdstud.ug.edu.pl (J.B.); zuzia.cyske@op.pl (Z.C.); grzegorz.wegrzyn@biol.ug.edu.pl (G.W.)

**Keywords:** cell activation, mucopolysaccharidoses, gene expression, transcriptomics

## Abstract

Although mucopolysaccharidoses (MPS), inherited metabolic diseases from the group of lysosomal storage diseases (LSD), are monogenic disorders, recent studies indicated that their molecular mechanisms are complicated. Storage of glycosaminoglycans (GAGs), arising from a deficiency in one of the enzymes involved in the degradation of these compounds, is the primary cause of each MPS type. However, dysfunctions of various cellular organelles and disturbance of cellular processes have been reported which contribute considerably to pathomechanisms of the disease. Here, we present a complex transcriptomic analysis in which all types and subtypes of MPS were investigated, with special emphasis on genes related to cell activation processes. Complex changes in expression of these genes were found in fibroblasts of all MPS types, with number of transcripts revealing higher or lower levels (relative to control fibroblasts) between 19 and over 50, depending on MPS type. Genes in which expression was significantly affected in most MPS types code for proteins involved in following processes, classified according to Gene Ontology knowledge database: cell activation, cell growth, cell recognition, and cell division. Levels of some transcripts (including *CD9, CLU, MME* and others) were especially significantly changed (over five times relative to controls). Our results are discussed in the light of molecular pathomechanisms of MPS, indicating that secondary and/or tertiary changes, relative to GAG storage, might significantly modulate cellular dysfunctions and contribute to molecular mechanisms of the disease. This may influence the efficacy of various therapies and suggests why various treatments are not fully effective in improving the complex symptoms of MPS.

## 1. Introduction

Mucopolysaccharidoses (MPS) are a group of lysosomal storage diseases occurring with a frequency of 1 in 40,000–50,000 live births [1]. They are caused by the lack or residual activity of glycosaminoglycan (GAG)-degrading enzymes, causing an accumulation of these polysaccharides in lysosomes, impairing cell functions. Eleven types/subtypes of this rare disease are distinguished due to the type of stored GAG(s) and the missing enzyme (Table 1). Symptoms of the disease usually appear between two and four years of age (depending on the type) and include organomegaly (mainly liver and spleen enlargement), thickening of the skin and subcutaneous tissue, which results in coarse facial features, as well as deformations within the skeleton, leading to disability of movement [1]. There may also be symptoms characteristic only for individual types of MPS, i.e., mental retardation (MPS I, II, III and VII), hyperactivity and aggression (MPS II and III), aortic valve regurgitation (MPS I, II, VI), corneal opacity (MPS I, IV, VI), and significant bone changes that can lead to paralysis in the subluxation mechanism between the atlas and the axis (MPS IV) [1]. The severity of the symptoms depend on many factors, among which the total lack or residual activity of lysosomal enzymes, the effectiveness of GAG synthesis [2] as well as environmental factors [3] or access to rehabilitation [4] and emergency assistance [5] can be mentioned.

Until recently, stored GAGs were considered to be the main, if not the only, cause of malfunctions of cells, and hence tissues, organs and the whole organism [6]. However, it turned out that enzyme replacement therapy, consisting of providing an active form of the missing enzyme to the organism, and resultant reduction in the level of GAGs, could not eliminate all the symptoms of the disease [7,8]. Thus, the question arises about aspects of MPS pathogenesis, other than GAG storage, at the molecular level. Recently published reports, focused on only some types of MPS, included descriptions of either proteomic studies, indicating changes in the levels of some important proteins (within the cell cytoskeleton or mitochondria), or electron microscopy studies on the number of intracellular vesicles [9,10,11,12]. Comprehensive studies, covering all types of MPS, on changes in various cellular processes not directly related to GAG metabolism, have started only recently, and have already published results concerning metabolic processes, apoptosis and processes related to human behavior [13,14,15]. However, many other important aspects of the functioning of MPS cells have never been tested in this aspect.

Activation, growth, division and recognition of cells are among the key aspects of homeostasis affecting the whole organism. The activation of cells, resulting from many signals reaching them, causes specific effects of their response, mainly adaptation to new environmental conditions (the lack of nutrients, growth factors, temperature changes, the appearance of reactive oxygen species and unfolded proteins, DNA and organelle damage or the appearance of pathogens or their toxins) influencing cell survival [16,17,18]. In the case of T or B cells, it allows the organism to defend itself by inducing a specific or non-specific response of the immune system [19]. In fibroblasts, production of extracellular matrix (ECM) structural proteins (collagen and elastin), adhesive proteins (laminin and fibronectin) or ground substance (GAG) is possible after fibroblasts activation, which has a great importance for wound healing and maintaining the continuity of the skin barrier and the entire connective tissue [20]. Growth and cell division, which are also the result of cell activation, are aspects of the growth of a young multicellular organism [21]. In adult organisms, these processes are crucial not for growth but for tissue renewal in continuous exchange (e.g., skin cells that divide only in the event of tissue damage) or regeneration (liver cells that retain the ability to divide throughout their lives) [22]. The processes of cell growth and division must be strictly regulated in order to maintain the correct proportions between different types of tissues in the organism [23,24]. In multicellular organisms, cell recognition processes are crucial to maintain the integrity of tissues and organs, as well as to control their functions. Various signal molecules and receptors are employed by cells (from proteins, through lipids to polysaccharides) to ensure proper recognition of neighbors and to coordinate appropriate cellular functions [25,26,27]. This applies to all types of cells, from fibroblasts to neurons. Defects in cell recognition can lead to serious disorders, including cancer, neurodegeneration and inflammatory diseases [28,29,30].

Due to the fact that changes in the processes of cell activation and their growth, division and recognition have never been considered as the basis for the pathogenesis of mucopolysaccharidoses, the purpose of this work was to comprehensively investigate changes in the transcriptomes of MPS fibroblasts, involving these processes in cells derived from patients with all types of the disease.

## 2. Results

As the disturbances of processes of cell activation, growth, division and differentiation have not been studied previously at the molecular level in the context of MPS pathogenesis, transcriptomic analyses of these processes in fibroblasts derived from patients with all types of MPS (type I, II, IIIA, IIIB, IIIC, IIID, IVA, IVB, VI, VII, IX), and healthy control (HDFa line) cells (Table 2), were performed. Cells were cultured under standard conditions, total RNA was isolated (in four biological, independent replicates) and sequenced using the RNA-seq technique. The number of readings in a single sample was at least 40,000,000.

The results indicated that the number of transcripts whose levels were significantly different (FDR < 0.1; *p* < 0.1) in MPS cells relative to healthy cells is most noticeable in the case of cell activation (GO: 0001775) process and ranges from 19 (for MPS VI) to as much as over 50 (for MPS I, IIIA, IIIB, IIIC, IVB and IX). The numbers of up- or down-regulated transcripts of genes related to cell growth (GO:0016049) and cell division (GO:0051301) processes were slightly lower, representing a minimum of about 10 (MPS II and VI for both processes) or exceeding 25 (for MPS IIIB and IIIC in cell growth process and for MPS VII in cell division process). Low numbers of altered transcripts (not exceeding 10) were also observed in cell recognition (GO:0008037) process (Table 3).

**Figure 1:**
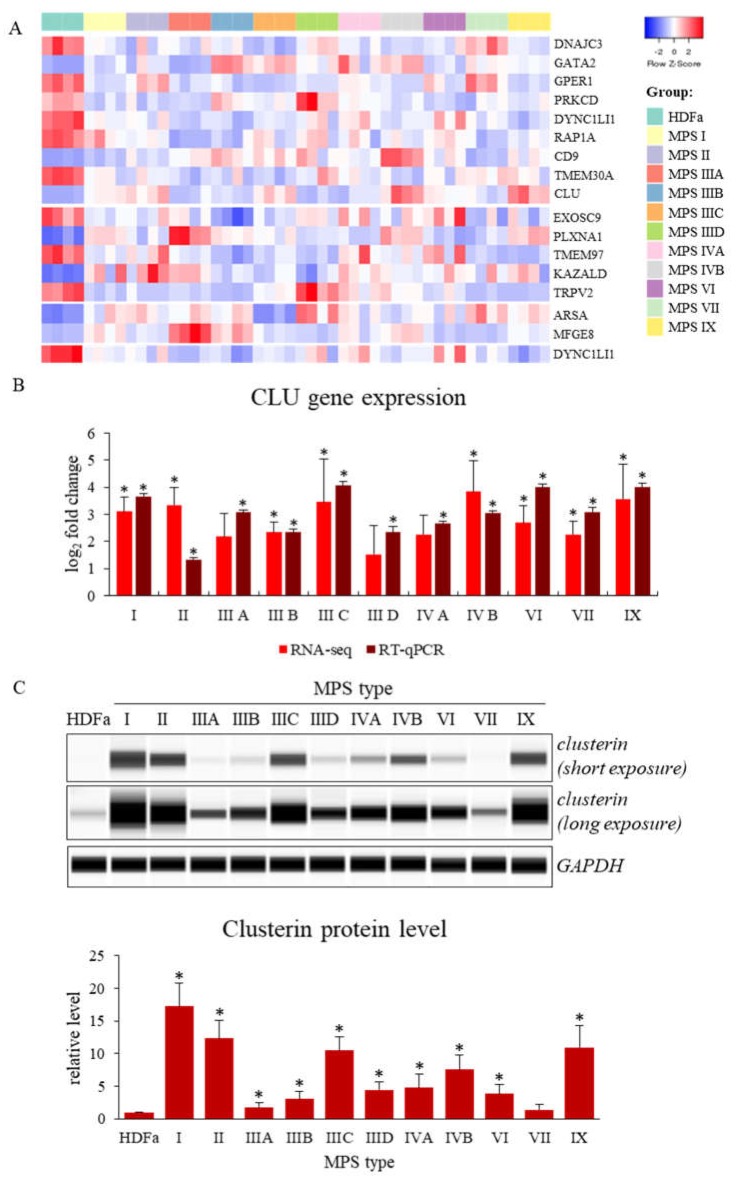
Genes with changed expression levels in at least six types/subtypes of MPS relative to HDFa cells which take part in cell activation, growth, division and recognition processes (according QuickGO database) (**A**), and levels of expression of the *CLU* gene analyzed by either RNA-seq and real-time PCR techniques (**B**) or Western-blotting (**C**) (representative blot and quantitative densitometric analysis). Presented results constitute the mean values of 4, 3 and 3 independent experiments, for RNA-seq, RT-qPCR, and Western-blotting, respectively). Error bars indicate standard deviation. Statistically significant differences compared to the results obtained for HDFa cells are indicated by an asterisk (*).

In the next stage of research, genes with expression significantly changed in most types of MPS were selected. Such an approach could indicate a possible mechanism of MPS pathogenesis, common to all types of this disease (the minimum number of MPS types in which the level of transcript was significantly changed was considered to be 6). The number of such genes varied between one (for cell division process) and nine (for cell activation process) (Table 4 and Figure 1A). The products of these genes are mainly involved in the regulation of intracellular signaling pathways and constitute receptors and other membrane proteins, but also genes whose products are heat shock proteins, proteins necessary to maintain cellular homeostasis, molecules involved in the proper functioning of the cytoskeleton or factors regulating gene expression can be found in this group.

Ten of these genes were down-expressed in MPS cells relative to healthy cells. They include genes of receptors, TRPV2 (transient receptor potential cation channel subfamily V member 2; *TRPV2* gene product) and estrogen receptor (the *GPER1* gene product), cellular kinases like tyrosine-protein kinase PRKCD (the *PRKCD* gene product) or components of cytoplasmic dynein complex, dynein cytoplasmic 1 light intermediate chain 1 (the *DYNC1LI1* gene product). However, some of the functionally important protein-encoding genes are up-regulated in cells taken from MPS patients. They include: plexin A1 (product of the *PLXNA1* gene), milk fat globule-EGF factor 8 protein, MFGE8 (product of the *MFGE8* gene), arylsulfatase A (products of the *ARSA* gene) or transcriptional factor GATA binding protein 2 (product of the *GATA2* gene). A particularly interesting gene undergoing increased expression in MPS is the *CLU* gene, encoding the clusterin protein (one of molecular chaperones). The results demonstrating altered *CLU* gene expression, obtained using the RNA-seq technique, were confirmed independently by RT-qPCR (Figure 1B). Moreover, increased levels of clusterin, the *CLU* gene product, in MPS cells were confirmed by Western-blotting (Figure 1C).

**Figure 2:**
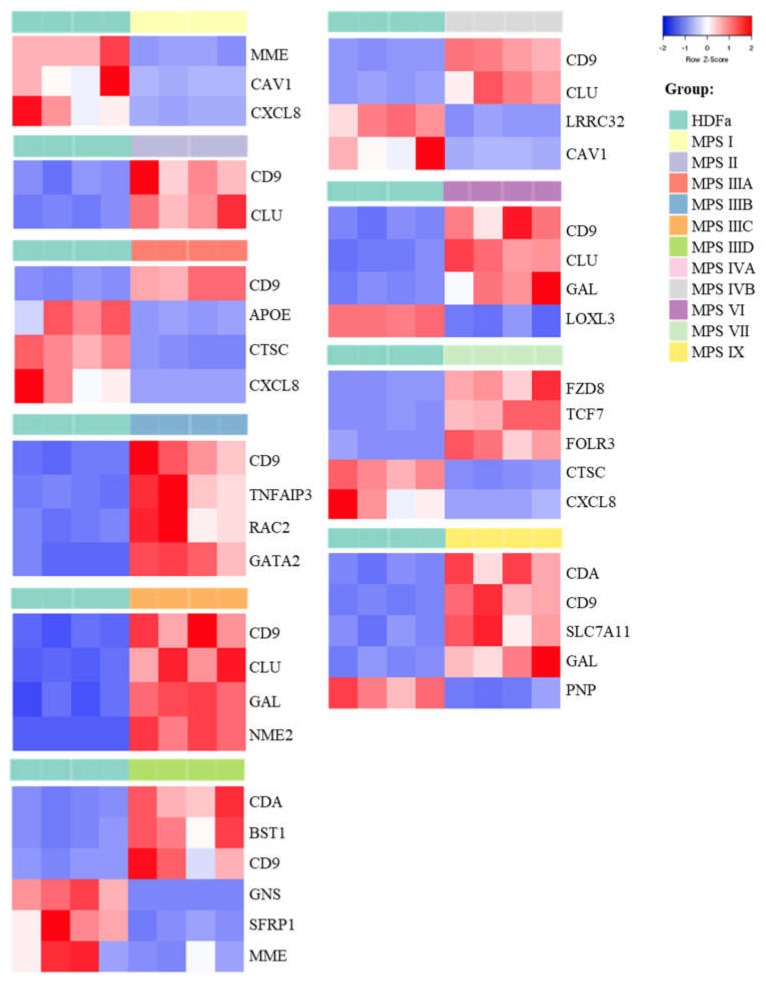
Heat-maps (created with Heatmapper programme) presenting genes particularly up- and down-regulated (FDR < 0.1; *p* < 0.1; log_2_ fold change (FC) > 2.5) in each type of MPS compared to HDF cells taking part in cell activation process.

**Figure 3:**
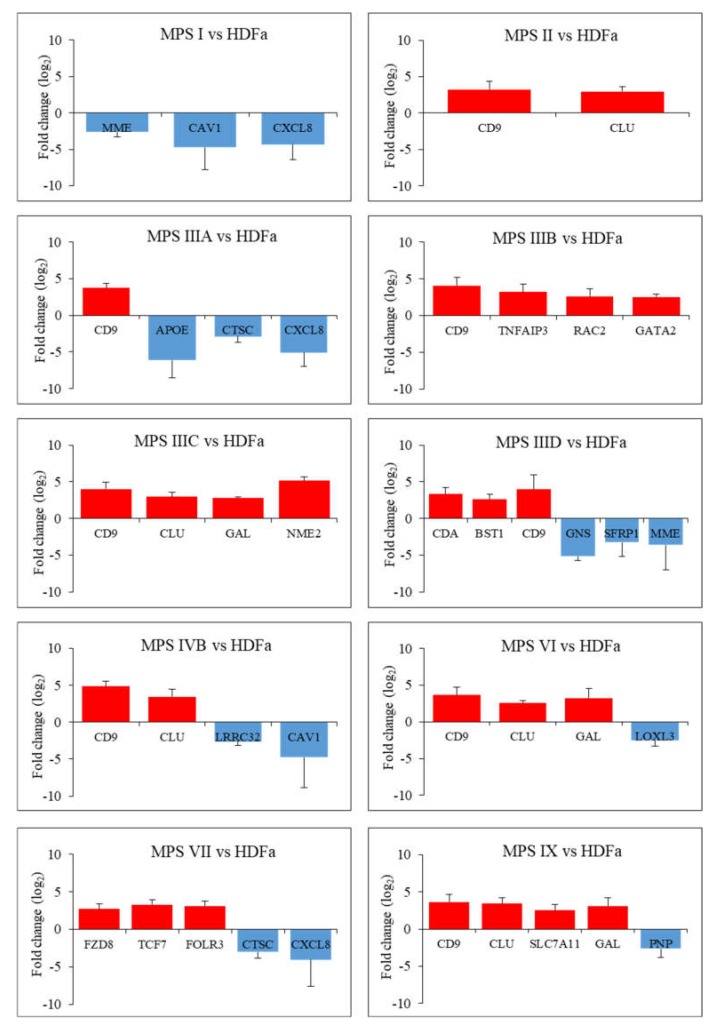
Diagrams presenting genes particularly up- and down-regulated (FDR < 0.1; *p* < 0.1; log_2_ fold change (FC) > 2.5) in each type of MPS compared to HDFa cells taking part in the cell activation process along with an indication of the exact log_2_ fold change (FC) value for each gene.

In the last stage of the study, genes whose fold changes in expression levels were the highest in MPS cells relative to control cells were selected, which indicates genes whose expression was at least 5.6 times up- or down-regulated (FDR < 0.1; *p* < 0.1; log_2_ fold change (FC) > 2.5). The number of genes whose expression is changed so strongly ranged from two (in the case of MPS II) to six (in the case of MPS IIID and VII) (Figure 2 and Figure 3 and Supplementary Figure S1).

**Figure 4:**
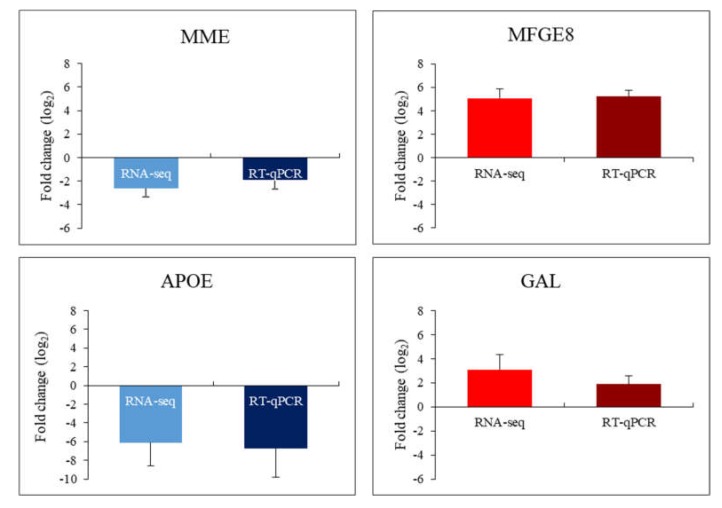
Diagrams presenting comparison of results indicating changes in the expression of selected genes by the RNA-seq and RT-qPCR methods (MME gene in MPS I; MFGE8 gene in MPS IIIA; APOE gene in MPS IIIA; GAL gene in MPS VI).

In this case, the vast majority of genes were overexpressed in patients’ cells relative to healthy controls. Among them, there is the previously described *CLU* gene encoding clusterin, which undergoes altered expression in many types of MPS (highly changed levels of expression were observed in MPS II, IIIC, IVB, VI and IX) as well as the *CD9* gene (encoding surface glycoprotein that forms complexes with integrins) which undergoes strong overexpression in MPS II, IIIA, IIIB, IIIC, IIID, IVB, VI and IX. The next strongly up-regulated genes, but with a lower number of MPS types, are *GAL*, encoding galanin protein (MPS type IIIC, VI and IX), *CDA* encoding cytosine nucleoside deaminase (MPS type IIID and IX), the already mentioned *GATA2* and others. Strongly miss-regulated genes also include examples of down-regulated genes, like the *MME* gene encoding the neprilysin protein and the *CAV1* gene encoding caveolin 1 protein which are characteristic for two types of MPS (MPS I, IIID and I, IVB, respectively) and examples of genes characteristic for one type of MPS, the *GNS* gene encoding glucosamine (*N*-acetyl)-6-sulfatase (MPS IIID), the *APOE* gene encoding apoliproteine E (MPS IIIA) and others. Results obtained for some of these genes, revealing the most pronounced changes in expression and potentially significantly influencing cell activation, were confirmed using RT-qPCR (Figure 4).

## 3. Discussion

Until recently, low residual activity or the lack of activity of one of the lysosomal enzymes (depending on the MPS type) was considered the only cause of MPS pathogenesis. Such enzyme deficiency leads to massive storage of GAG in cells, damaging the functions of entire tissues and organs [1]. The easiest way of treatment seems to be supplementation of the cells with the ‘missing’ enzyme, which therapy should reverse the negative effects of GAG accumulation [6].

However, it turns out that enzyme replacement therapy (ERT) does not always give the expected results in the form of the elimination of all symptoms. Admittedly, the administration of the enzyme significantly improves the condition of patients with mild forms of MPS I [31], II [32] and VI (in which symptoms affect somatic tissues); however, this treatment is problematic for types in which the symptoms affect the central nervous system, i.e., type I and II (severe forms), as well as IIIA, IIIB, IIIC, IIID and VII due to the failure of enzyme to cross the blood–brain barrier or to reach the skeletal system [33,34]. These results suggest either the complexity of GAG-induced cell disorders that once developed cannot be reversed or previously unknown aspects of disease pathogenesis that were not taken into consideration in this therapy. The purpose of this work was, therefore, to perform a transcriptomic analysis of one of the key cellular processes, cell activation, but also cell growth, recognition and division, in patients with all types of MPS.

To date, excessive cell activation has been described mainly in the context of the immune system, i.e., mast cells, resulting in the abnormal release of mediators of these cells, and thus, affecting functions in potentially any organ system [35], or T and B cells, which results in severe autoimmune diseases such as rheumatoid arthritis [36], common variable immunodeficiency [37] or systemic lupus erythematosus [38]. Immune system cells are not the only type of cells whose excessive activation can have huge consequences for the organism. Activation of fibroblasts, the main cells that are part of the connective tissue, is an important process in the context of their secreted extracellular matrix components such as ground substance, adhesive proteins (fibronectin and laminin) and structural proteins (collagen and elastin), whose proper proportion is necessary to maintain rigidity/elasticity of the tissue. The activation of fibroblasts plays an important role not only in the maintenance and reabsorption of extracellular matrix, but also in inflammation and wound healing. However, in some cases this activation becomes uncontrolled, causing a pathological tissue fibrotic response. Such fibrosis plays a significant role in most cases of organ failure and is the cause of systemic sclerosis, idiopathic pulmonary fibrosis, cirrhosis, kidney fibrosis and cardiac fibrosis [20,39].

Disturbances in the signals that control cell growth and division can also result in serious symptoms, such as hypertrophy, that prevent proper functions of organs. Although the concepts of hypertrophy and hyperplasia are different, they are most often controlled by the same regulations at the molecular level [40]. Excessive cell growth can be caused by increased production of some proteins like growth factors (IGF, FGF, TGF), activation of some signaling pathways (PI3K and AKT-dependent) and activation of some transcription factors (GATA4, MEF2, NFAT). The effects of these phenomena can be observed in the case of myostatin-related muscle hypertrophy [41] or cardiac hypertrophy [42].

Cell recognition disorders, especially important in the context of the immune system, can also lead to negative effects. Mutations in genes encoding some receptors involved in cell-cell interactions (e.g., TLRs) cause these receptors to lose their ability to bind to polysaccharides, lipids or proteins in the membrane of unicellular pathogens, resulting in a lack of response to infections by the immune system, allowing for the rapid development of the disease [43]. Fibroblasts are also involved in the immune system’s response to inflammation through cellular interactions. Inflammatory cells often accumulate in the extravascular connective tissue, and reaching lymphocytes to such a place and stopping it there is dependent on the direct interaction of lymphocytes with fibroblasts. In the case of disorders of these interactions, tissue destruction can easily occur due to the appearance of chronic inflammation [44].

In summary, all the processes listed above are crucial for the proper functioning of the organism. Changes in the expression of key genes involved in these processes can result in significant changes in their course or performance. A comprehensive assessment of the transcriptomic profile of genes involved in these processes in the context of MPS pathogenesis has never been performed.

In our study, among genes in which expression level changes in relation to healthy cells there were those related to all the processes mentioned in this article, while the vast majority of them concerned cell activation. This suggests large changes in this phenomenon in the case of MPS (Table 3). This number is particularly high for MPS IVB, as well as MPS IIIA, IIIB and IIIC. Indeed, excessive collagen deposition in the myocardium and significant left ventricular fibrosis were recognized in the MPS IIIB model mice, which clearly indicates cell dysfunction [45]. Similar cases, but with hepatic fibrosis, were described in MPS type I, II and III [46]. It is worth noting that MPS type IVB itself is not phenotypically similar to MPS III, however, another mutation of the same gene (*GLB1*), encoding beta-galactosidase, is also the cause of GM1 gangliosidosis, in which the clinical picture is similar to that found in MPS III [47].

Cell growth disorders can also be represented as part of the MPS pathogenesis. One of the first symptoms of the disease is hepatomegaly or hepatosplenomegaly. It is currently believed that these phenomena are caused by abnormal cell enlargement through GAG accumulation [1]. The causes of this pathological process have never been sought in the unrestrained growth of liver and spleen cells, which can also be caused by GAG storage, by affecting the expression of factors that regulate cell growth.

Cell recognition depends primarily on receptors present on their membranes, thanks to which they receive a signal from the external environment. Little is known about disturbances in the functioning of the receptors themselves in MPS, despite the fact that the molecular mechanisms of receiving signals by receptors leading to specific pathological phenomena in cells, are increasingly known. The examples are pain (ATER and ETBR receptors) [48], innate immunity (TLR4 receptor) [49] or disorders of bone and heart development and function (Ib BMP and FGFR2 receptors) [50]. To date, several examples of GAG interaction with various receptors have been described, which consequently can inhibit or stimulate their activity (up-regulated TGF-β signaling, down-regulated BMP signaling and others) [50]. Disruption of receptor activity is certainly reflected in cell recognition processes.

In the context of MPS pathology, the detected changes in gene expression affecting many types of this disease are very important because they can indicate a potential common mechanism characterizing all types of MPS (Table 4 and Figure 1A).

**Figure 5:**
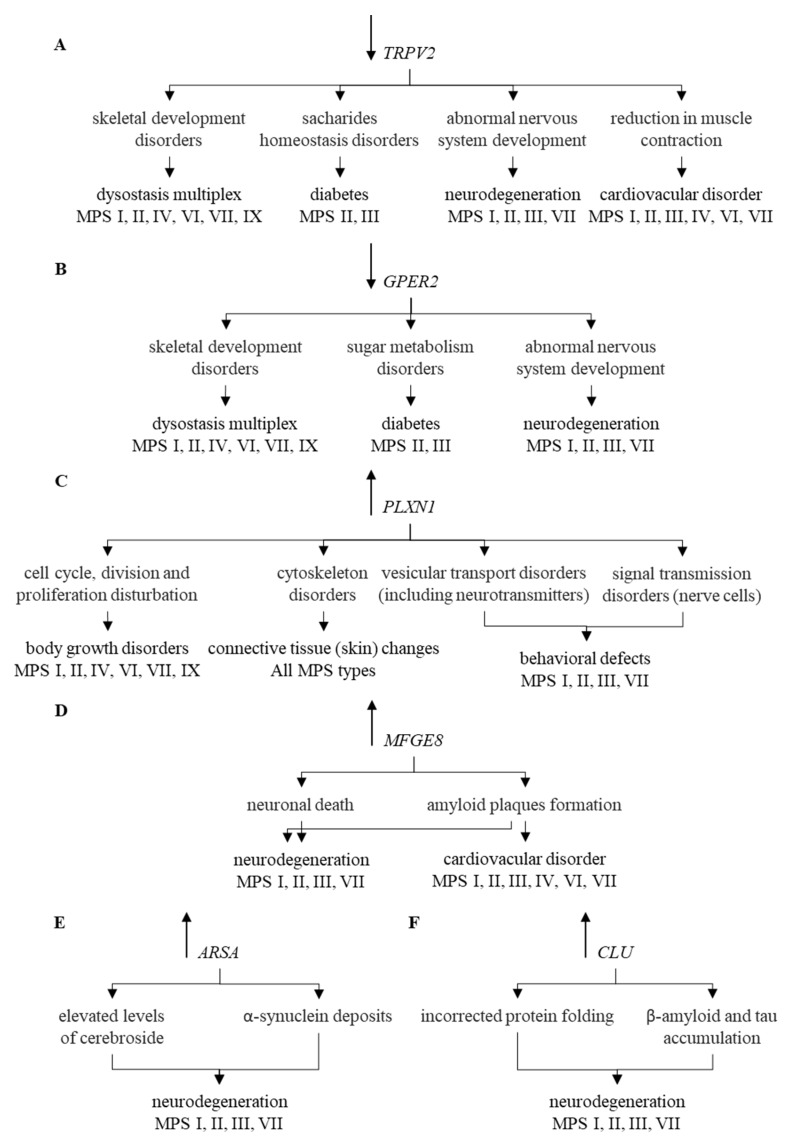
Correlation of changes in the expression of individual genes (*TRPV2* (**A**), *GPER2* (**B**), *PLXN1* (**C**), *MFGE8* (**D**), *ARSA* (**E**), and *CLU* (**F**)) and their functions with symptoms occurring in particular types of MPS. Symbols ↑ and ↓ near gene names indicate elevated and decreased levels of corresponding transcripts in MPS vs. control cells, respectively.

Modulation of expression of such genes may contribute to explanation of mechanisms of appearance of at least some symptoms characteristic for MPS. These genes are exemplified by *TRPV2* whose changed expression can lead to diabetes [51,52], bone disorders [53], and cardiovascular symptoms [54,55,56,57]. All of these disorders occur in MPS, and TRPV2-mediated mechanisms of their appearance are depicted in Figure 5A.

Another example is *GPER1*, coding for the estrogen receptor, whose functional disturbances result in neuronal dysfunctions [58,59], glucose intolerance [60,61], increased blood pressure [62], changed bone mineral density [63,64], abnormal joint mobility, and osteoarthritis [65,66]. Neuroprotection mediated by this protein has also been reported [67]. Thus, one may link these effects to symptoms observed in MPS patients, as depicted in Figure 5B. The *PLXNA1* gene codes for plexin, influencing functions of the cytoskeleton after binding of its ligand, semaphoring [68,69]. Changed expression of this gene may result in improper development of various tissues and organs [70], including nervous system and connective tissue [71,72]. This resembles connective tissue disorder [73], including the skin barrier [74], occurring in MPS. Plexin-related changes have been connected to various impairments in functions of central nervous system [75,76,77,78] which are also common in some MPS types, as indicated in Figure 5C. Dysregulation of the *MFGE8* gene has been linked to neurodegenerative processes dependent on the accumulation of amyloid [79,80,81,82], and a similar phenomenon has been described in MPS [83,84,85], which is presented in Figure 5D.

**Figure 6:**
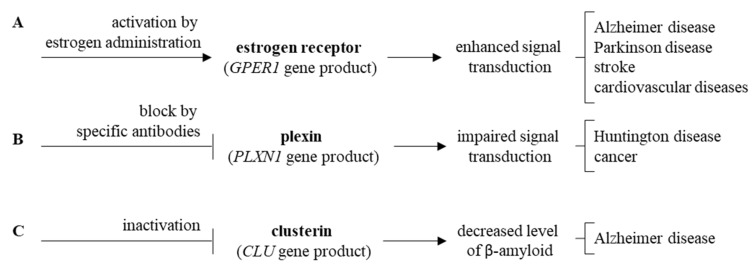
Proposed new therapeutic strategies targeting estrogen receptor (**A**), plexin (**B**), and clusterin (**C**).

Impaired *ARSA* gene functions, encoding arylasulfatase A, results in disturbed cerebroside metabolism which affects functions of neurons, thus, leading to neuronopatic symptoms resembling those occurring in MPS [86,87,88,89,90,91,92] (Figure 5E). Finally, elevated levels of clusterin, encoded by the *CLU* gene, were demonstrated in various neurodegenerative diseases [93,94,95,96,97,98,99,100,101,102,103], and enhanced *CLU* expression was evident in MPS cells (compare Figure 5F). It is also worth mentioning that the estrogen receptor, plexin and clusterin or their genes might be considered as potential therapeutic targets for novel drugs, as depicted in Figure 6.

Modulation of the expression of some of the above-mentioned genes, or other genes from a similar group, have already been indicated in the case of MPS, e.g., *TRPV2*, *PLXNA1* or *CLU*. However, the rest of them are completely new aspects of MPS pathogenesis. The question remains whether these changes are a direct consequence of the accumulation of GAG(s) in cells by binding various transcription factors, non-coding RNAs and/or other molecules that enable/prevent the expression of individual genes, which affects the disturbance of many cell processes, or are they already independent of the GAG level changes that cannot be reversed even after the use of enzyme replacement therapy or a therapy involving the reduction of substrate synthesis.

In addition to the group of symptoms common to all types of MPS, there are symptoms specific to each type. Therefore, in this work, attention was also paid to genes in which expression levels are changed significantly, i.e., more than 5-fold for each type of MPS separately (Figure 2 and Figure 3 and Supplementary Figure S1). Apart from the *CLU* gene, another gene which expression is increased in most MPS types is *CD9*. The *CD9* gene product, which is a surface glycoprotein, physiologically binds integrins, which leads to the activation of many processes important for the cell. The problem is that heparan sulfate, stored in the case of MPS I, II, III and VII, has the ability to bind to integrins [104] which may prevent them to bind to CD9 and promote signal transduction. It cannot be ruled out that the situation is similar for other GAGs, and the increase in CD9-encoding gene expression is the cell’s response to the need for downstream stimulation of this protein. Decreased expression of this gene has already been indicated in the case of nervous tissue in the MPS type VII mouse model. The authors of the study pointed to the participation of this gene in the late maturation of myelinating oligodendrocytes and neurons as a consequence of its expression disorders [105]. Disturbance in expression of genes which led to inappropriate composition of the myeline, has also been reported in the MPS I dog model [106].

The neprilysin protein (the *MME* gene product) is known primarily for its increased expression in one of the pre-B phenotype of acute lymphocytic leukemia, thanks to which it owes its second name, common acute lymphoblastic leukemia antigen (CALLA) [107]. There are also reports indicating cases of a decrease in its level in ulcerative enteritis [108] or heart failure [109], and mutations occurring in some diseases, such as autosomal-recessive Charcot-Marie-Tooth disease type 2, characterized by deformation of the feet and the disappearance of some muscles and reflexes [110], and inherited peripheral neuropathies [111]. Furthermore, elevated levels of some neprilysin substrates (glucagon and insulin) have been described as the main cause of diabetes [112], and one of the symptoms associated with sickle cell disease (substance P) [113]. Another connection with Alzheimer’s disease appears very interesting because many specialists working on this disease have observed a decrease in neprilysin levels in patients and in animal models of this neurological disease [114,115,116]. It turns out that another substrate for neprilysins (next to glucagon, insulin, substance P and many others) is amyloid, thus, lowering its level promotes the formation of its deposits and the formation of amyloid plaques [116,117,118].

As mentioned earlier, amyloid deposits have also been found in some cases of MPS, so reduced expression of the *MME* gene can contribute to this phenomenon. Furthermore, increasing neprilysin activity is considered as an attempt to support the treatment of Alzheimer’s disease. Delivery of active protein into the organism, however, requires the use of vectors, which is accompanied by the same challenges as current work on gene therapy (stimulation of the immune response, secondary toxicity and low efficiency) [119,120]. Despite this, attempts are being made to input the active form of neprilysin into the brain [121], intraperitoneally [122] and intramuscularly [123], and all these attempts seem to be successful in terms of reducing deposits of amyloid. Perhaps these strategies will also be worth considering when combined therapy for MPS is developed, mainly for types that affect the central nervous system.

Patients with MPS often complain of pain caused by various symptoms (tissue damage, inflammation, etc.) It is not entirely clear whether an increase in the *GAL* gene expression can contribute or, on the contrary, is the response to these phenomena. These doubts result from the fact that galanin has a dual effect on nociception, depending on which receptor is activated by it, GalR1 (inhibitory effect) or GalR2 (stimulant effect) [124,125]. Similarly, the previously mentioned substance P, which is also a pain mediator, may also affect pain perception. This is due to the fact that substance P probably does not undergo cleavage due to low levels of galanin (as a result of low expression levels of the *MME* gene), which should physiologically deactivate it [48]. An increase in the *GAL* gene expression and galanin levels has already been found in some diseases, among which Alzheimer’s disease is again at the forefront. Post-mortem brain tissue analyzes in patients with Alzheimer’s disease indicated more than 200% expression level of this gene relative to control [126]. However, the results obtained by many teams on the role of galanin in the pathogenesis of this disease are ambiguous. Some studies indicated the presence of previously unknown abnormal fibers in the nerve tissue, especially rich in galanin, which seems to innervate cholinergic neurons (GAL-containing fibers). One hypothesis considers the negative effect on the survival of such hyper-innervation neurodes due to blocking acetylcholine access to these neurons [127]. The other hypothesis was proposed by Ding et al. (2006), who suggested that over-expression and hyper-innervation of neurons promotes the survival of cells affecting the protection of the neuron against amyloid toxicity [128]. There is currently no information about GAL-containing fibers or hyper-innervation in the case of MPS. This issue is undoubtedly worth studying, and searching for more mechanisms of the pathogenesis of the disease is substantiated. However, even if these facts were true in the case of MPS, due to an unknown role of GAL in degeneration/protection of neurons, the use of GAL as a therapy target for MPS would be quite risky.

Another example of a gene that is a subject to reduced expression is the *APOE* gene, encoding apoliprotein E, which is mainly responsible for transporting one of the key cell components, cholesterol. It is known that disturbance of the homeostatic state, which is correlated to apolipoprotein E function, is associated with cardiovascular diseases and is often attributed to nutrition habits and lifestyle [129]. Clinical and experimental evidence suggests that changes in cholesterol metabolism may also play an important role in carcinogenesis and tumor development [130,131]. Unfortunately, knockout of *ApoE*^−/−^ mice has long been known to cause severe atherosclerosis and neurological disorders, including memory and learning defects [132,133]. Mutations in the *APOE* gene are associated with familial hypercholesterolemia associated with premature cardiovascular disease [134]. Reports about the positive effect of lowering APOE levels on the body, including age-related bone healing, are also more and more frequent [135]. Regarding MPS, changes in lipid metabolism and cholesterol levels [136] or its location [137] have been observed in MPS IIIA mice. However, different results were obtained by various teams, as some studies suggested that the risk of hypercholesterolemia and diabetes in MPS does not exceed the average population risk [51].

The *NME2* gene, encoding one of the nucleoside-diphosphate kinase subunits, is involved in maintaining normal cell metabolism and signal transduction in the cell by participating in many chemical reactions. To date, most of the described cases of implications of nucleoside-diphosphate kinase in diseases were proposed to result from inhibition of its activity, which occurred in Alzheimer’s disease, Down syndrome [138] and diabetic retinopathy [139]. However, several reports on increased *NME2* gene expression have also been reported in adenocarcinoma of the prostate [140], solid tumors [141] and stroke [142]. Moreover, an increase in expression of this gene has also been shown in sarcolemmal membranes of failing human myocardium causing a decrease in adenylate cyclase activity [143], which again suggests its involvement in the cardiovascular problem. This protein can interact with other enzymatic proteins, such as AMPK, or ion channels, such as CFTR [144,145], which may be important in cystic fibrosis, and with graphene oxide [146], and may adversely affect heart failure. Modulations of the expression of this gene or of the activity of the corresponding protein have never been described in the MPS catalog.

To avoid a potential problem with interpretation of the results arising from using fibroblasts derived from persons at different ages, we have analyzed previous reports addressing this issue. In fact, age-related expression of genes in fibroblasts has been reported previously [147,148,149]. Nevertheless, among the transcripts with the most efficiency changed levels in MPS cells, there were no evidently age-related genes. The only exception might be *TMEM97*. Although age-related expression of this gene was not reported, such a relation has been discovered for *TMEM47* [149], a gene from the same family. Therefore, we assume that our conclusions are not significantly affected by age-related changes in gene expression. One should also consider some limitations of this study. Only one line of each MPS type was investigated, only fibroblasts were studied, and protein levels were not determined for most of genes. However, the facts that expressions of most of genes were changed in the same direction in most MPS types, and that changes in levels of the selected protein (clusterin) corresponded to changes in mRNA levels, suggest that observed changes may reflect actual properties of MPS cells, at least to some extent.

## 4. Materials and Methods

### 4.1. Cell Lines and Cell Cultures

Fibroblasts derived from patients with all types of MPS (I, II, IIIA, IIIB, IIIC, IIID, IVA, IVB, VI, VII, IX) and the control HDFa (Human Dermal Fibroblasts, adult) cell line, were purchased from the Coriell Institute (Table 2). Cells were cultured in the DMEM medium, supplemented with 10% Fetal Bovine Serum (FBS), and in the presence of antibiotics under standard conditions at 37 °C, 95% humidity with an atmosphere saturated with 5% CO_2_.

### 4.2. RNA Isolation and Purification

First, 5 × 10^5^ cells were seeded in 10-cm diameter plates and allowed to attached overnight. The next day, cells were lysed in the lysis buffer containing guanidine isothiocycanate and beta-mercaptoethanol, and homogenized with the QIAshredder column. RNA was isolated using the RNeasy Mini kit (Qiagen, Hilden, Germany) using Turbo DNase (Life Technologies, Carlsbad, CA, USA) following the manufacturer’s instructions. The quality of the isolated RNA (3 μg, 50 ng/µl, RIN > 6.8) was determined in the Agilent 2100 Bioanalyzer System using RNA Nano Chips (Agilent Technologies, Santa Clara, CA, USA).

### 4.3. RNA-Seq Analysis

Illumina TruSeq Stranded mRNA Library Prep Kit was used to create cDNA libraries that were then sequenced using HiSeq4000 (Illumina, San Diego, CA, USA) with the following parameters: PE150 (150bp paired-end) and 40 million readings in each sample. FastQC version v0.11.7 was used to assess the quality of analyzes, and the raw data was deposited with NCBI (NCBI Sequence Read Archive (SRA): PRJNA562649). Obtained data were mapped to the human reference genome GRCh38 from the Ensembl database using the Hisat2 v. 2.1.0 program. The Cuffquant and Cuffmerge programs in version 2.2.1 and the GTF Homo_sapiens.GRCh38.94.gtf file from the Ensembl database were used to examine transcripts FPKM (Fragments Per Kilobase of transcript per Million fragments mapped) levels and changes in the level of gene expression. Transcript annotation and classification was performed using the QuickGO database.

### 4.4. Reverse Trascription qPCR

RT-qPCR experiments were performed for selected genes. Total RNA was reverse transcribed into cDNA using the NG dART RT kit (EURx: Molecular Biology Products, Gdansk, Poland), following the manufacturer’s instructions. qPCR was carried out using LightCycler® 480 SYBR Green I Master [Roche, Basel, Switzerland] in Roche LightCycler 480 equipment. The fold change in the expression of individual genes between different cell lines was determined using the 2−ΔΔ*C*(T) method. GAPDH served as a reference gene for the performed analyzes. The sequences of the primers used in RT-qPCR assay are shown in Supplementary Table S1.

### 4.5. Western Blotting

Clusterin levels were determined using Western blotting with the WES system (WES - Automated Western Blots with Simple Western; ProteinSimple, San Jose, California, USA), with 12–230 kDa Separation Module (#SM-W003) and Anti-Rabbit Detection Module (#DM-002), as described previously [150]. Primary Anti-clusterin antibodies were used to determine levels of the *CLU* gene product, and anti-GAPDH antibodies were used as an internal control. Antibodies (rabbit mAb #34642 and #5174) were purchased from Cell Signaling Technology (Leiden, The Netherlands).

### 4.6. Statistical Analysis

In RNA-seq, one-way analysis of variance (ANOVA) on log_2_ (1 + x) values was used to examine statistically significant changes among samples with normal distribution and which have normal continuous distribution (cell line type was the independent variable in this test), and the Benjamini-Hochberg method was used to estimate the false discovery rate (FDR). The significance of the changes between two groups was analyzed using post hoc Student’s t-test with Bonferroni correction (this test, rather than Tukey, was chosen on the basis of the analysis of distribution of variables, and considering the fact that the Tukey test allows the identification of the highest difference, skipping smaller ones if they co-exist). All statistical analyzes were performed using R v3.4.3 software. In RT-qPCR, one-way analysis of variance (ANOVA) was used to determine significant differences in the levels of gene expression between the tested cell lines.

## 5. Conclusions

In conclusion, the cases of modulation of the expression of important genes influencing the proper functioning of cells and the whole organism indicated in this work are in most cases completely new aspects of MPS pathogenesis. Moreover, these results may point to the basis of various dysfunctions appearing in this disease in the form of severe symptoms with an undiagnosed mechanism. On the other hand, the detected changes may also constitute new markers to monitor the therapies, which are missing in the case of MPS, or constitute new targets for possible combined therapies, which are needed especially for types for which the currently used therapies are ineffective or only partially effective.

## Figures and Tables

**Table 1:** Types/subtypes of MPS (based on [1]).

MPS Type	Stored GAG(s)	Defective Enzyme
MPS I	HS, DS	α-L-iduronidase
MPS II	HS, DS	2-iduronate sulfatase
MPS IIIA	HS	*N*-sulfoglucosamine sulfhydrolase
MPS IIIB	HS	α-*N*-acetylglucosaminidase
MPS IIIC	HS	Acetyl-CoA:α-glycosaminide acetyltransferase
MPS IIID	HS	*N*-acetylglucosamine 6-sulfatase
MPS IVA	KS, CS	*N*-acetylgalactosaminide 6-sulfatase
MPS IVB	KS	β-galactosidase-1
MPS VI	DS	*N*-acetylgalactosamine 4-sulfatase
MPS VII	HS, DS, CS	β-glucuronidase
MPS IX	HA	Hyaluronidase-1

Abbreviations: HS, heparan sulfate; DS, dermatan sulfate; KS, keratan sulfate; CS, chondroitin sulfate; HA, hyaluronic acid.

**Table 2:** Characteristics of cells derived from MPS patients [according to Coriell Institute description].

Cell Line	Race	Sex	Age (Years)	Locus of Mutated Gene	Mutation Type
MPS I	Caucasian	F	1	*IDUA*, 4p16.3	Homozygote p.Trp402Ter/p.Trp402Ter
MPS II	Caucasian (ethnicity: Haitian)	M	3	*IDS*, Xp28	Hemizygote p.His70ProfsTer29
MPS IIIA	Caucasian	F	3	*SGSH*, 17q25.3	Complex heterozygote p.Glu447Lys/p.Arg245His
MPS IIIB	Caucasian	M	7	*NAGLU*, 17q21	Homozygote p.Arg626Ter/p.Arg626Ter
MPS IIIC	unknown	M	8	*HGSNAT*, 8p11.1	ND
MPS IIID	Asian Indian	M	7	*GNS*, 12q14	Homozygote p.Arg355Ter/p.Arg355Ter
MPS IVA	Caucasian (ethnicity: Mexican)	F	7	*GALNS*, 16q24.3	ND
MPS IVB	Caucasian	F	4	*GLB1*, 3p22.3	Complex heterozygote p.Trp273Leu/p.Trp509Cys
MPS VI	Black	F	3	*ARSB*, 4q14.1	ND
MPS VII	African American	M	3	*GUSB*, 7q21.11	Complex heterozygote p.Trp627Cys/p.Arg356Ter
MPS IX	unknown	F	14	*HYAL1*, 3p.21.3	ND

Abbreviations: F, female; M, male; ND, not determined.

**Table 3:** Number of up- and down-regulated transcripts related to cell activation, growth, division and recognition processes (according to the QuickGO database) in different types of MPS relative to HDFa cells.

Transcripts	No. of Transcripts with Changed Expression Levels in MPS vs. HDFa Line										
	I	II	IIIA	IIIB	IIIC	IIID	IVA	IVB	VI	VII	IX
	Cell activation (GO:0001775)										
Up-regulated	18	16	19	33	24	16	8	29	8	25	22
Down-regulated	35	11	38	27	32	18	10	47	11	20	31
Total	53	27	57	60	56	34	18	76	19	45	53
	Cell growth (GO:0016049)										
Up-regulated	7	2	9	15	14	7	4	11	6	10	14
Down-regulated	11	0	14	10	11	9	4	8	2	7	9
Total	18	2	23	25	25	16	8	19	8	17	23
	Cell division (GO:0051301)										
Up-regulated	9	2	7	6	6	5	2	10	1	10	2
Down-regulated	11	6	8	14	11	19	0	7	1	16	18
Total	18	8	15	20	17	24	2	17	2	26	20
	Cell recognition (GO:0008037)										
Up-regulated	3	2	5	3	4	4	2	5	0	3	3
Down-regulated	3	1	0	1	3	2	2	1	3	3	3
Total	6	3	5	4	7	6	4	6	3	6	6

**Table 4:** Values of fold change (FC) of genes with changed expression levels in at least six types/subtypes of MPS relative to HDFa cells (only in case of a significant change).

Gene	Value of Fold Change (FC) of Transcript Level in MPS Types										
	I	II	IIIA	IIIB	IIIC	IIID	IVA	IVB	VI	VII	IX
	Cell activation										
*DNAJC3*	0.6 ± 0.0	-	-	0.7 ± 0.1	0.6 ± 0.0	-	0.7 ± 0.1	0.6 ± 0.0	0.7 ± 0.1	0.9 ± 0.1	0.7 ± 0.0
*GATA2*	2.6 ± 0.5	-	-	5.8 ± 0.9	4.7 ± 0.7	3.4 ± 0.6	-	4.9 ± 0.6	-	3.5 ± 0.5	3.1 ± 0.3
*GPER1*	0.4 ± 0.1	-	0.4 ± 0.1	-	0.3 ± 0.1	0.4 ± 0.0	0.5 ± 0.1	0.4 ± 0.1	-	-	0.3 ± 0.1
*PRKCD*	0.4 ± 0.1	-	0.4 ± 0.1	-	0.6 ± 0.1	-	0.6 ± 0.1	0.5 ± 0.1	0.4 ± 0.1	-	-
*DYNC1LI1*	0.7 ± 0.0	0.7 ± 1.9	0.6 ± 0.0	0.6 ± 0.1	0.7 ± 0.0	-	-	0.7 ± 0.0	-	0.7 ± 0.1	0.6 ± 0.1
*RAP1A*	-	0.7 ± 0.0	0.6 ± 0.0	0.7 ± 0.1	-	-	-	0.7 ± 0.0	0.8 ± 0.0	0.7 ± 0.1	0.7 ± 0.0
*CD9*	-	9.3 ± 3.3	13.5 ± 2.1	17.1 ± 4.6	15.9 ± 3.7	15.5 ± 7.7	-	28.9 ± 4.0	12.4 ± 3.8	-	12.8 ± 3.6
*TMEM30A*	-	0.7 ± 0.1	0.6 ± 0.0	-	0.6 ± 0.0	-	0.7 ± 0.1	0.6 ± 0.1	-	0.8 ± 0.0	-
*CLU*	6.4 ± 0.7	7.9 ± 1.6	-	3.4 ± 0.7	7.8 ± 1.6	-	-	11.0 ± 3.2	6.0 ± 0.8	4.2 ± 1.4	10.9 ± 1.4
	Cell growth										
*EXOSC9*	0.2 ± 0.1	-	0.5 ± 0.2	0.3 ± 0.2	0.2 ± 0.1	0.3 ± 0.1	0.3 ± 0.3	0.5 ± 0.2	-	-	0.3 ± 0.2
*PLXNA1*	1.8 ± 0.0	-	2.3 ± 0.3	1.7 ± 0.1	1.6 ± 0.1	-	-	1.9 ± 0.1	-	-	1.8 ± 0.1
*TMEM97*	0.4 ± 0.1	-	-	0.2 ± 0.1	0.4 ± 0.1	0.3 ± 0.1	-	0.5 ± 0.1	-	0.3 ± 0.1	0.2 ± 0.1
*KAZALD1*	-	-	3.9 ± 0.5	2.5 ± 0.4	2.6 ± 0.5	-	3.7 ± 0.5	2.9 ± 0.3	3.3 ± 0.6	-	-
*TRPV2*	0.1 ± 0.1	-	0.2 ± 0.1	0.1 ± 0.0	0.4 ± 0.1	-	-	0.4 ± 0.1	-	0.1 ± 0.1	0.1 ± 0.0
	Cell recognition										
*ARSA*	2.0 ± 0.4	2.3 ± 0.3	2.2 ± 0.3	2.1 ± 0.3	-	2.8 ± 0.6	2.0 ± 0.3	1.8 ± 0.2	-	2.6 ± 0.4	2.2 ± 0.2
*MFGE8*	6.2 ± 1.2	3.1 ± 0.9	14.9 ± 3.1	8.3 ± 1.8	3.2 ± 0.9	-	-	6.8 ± 1.0	-	-	4.2 ± 0.5
	Cell division										
*DYNC1LI1*	0.7 ± 0.0	0.7 ± 0.1	0.6 ± 0.0	0.6 ± 0.1	0.7 ± 0.0	-	-	0.7 ± 0.0	-	0.7 ± 0.1	0.6 ± 0.1

## Supplementary Materials

Supplementary materials can be found at https://www.mdpi.com/1422-0067/21/9/3194/s1. Figure S1: Heat-maps (created with Heatmepper programme) and diagrams presenting genes particularly up-and down regulated (FDR < 0.1; *p* < 0.1; log_2_ fold change (FC) > 2.5) in each type of MPS compared to HDFa cells taking part in cell growth, recognition and division processes along with an indication of the exact log_2_ fold change (FC) value for each gene; Table S1. Primer sequences used for real-time PCR in studies on expression of selected genes (The Primer Bank MGH-PGA was used to determine sequences of primers).

## Abbreviations

ERT	enzyme replacement therapy
ECM	extracellular matrix
FDR	false discovery rate
FC	fold change
GAG(s)	glycosaminoglycan(s)
LSD	lysosomal storage disease
MPS	mucopolysaccharidosis
