# Supplementary material for: Transcriptomic Changes Related to Cellular Processes with Particular Emphasis on Cell Activation in Lysosomal Storage Diseases from the Group of Mucopolysaccharidoses
Source: Int J Mol Sci. 2020 Apr 30;21(9):3194. doi: 10.3390/ijms21093194 (PMC7246638; doi:10.3390/ijms21093194)
Supplement: Supplementary file 1 [file ijms-21-03194-s001.pdf]

Supplementary material

Table S1. Primer sequences used for real-time PCR in studies on expression of selected genes (The PrimerBank MGH-PGA was used to determine sequences of primers).

Gene	Forward primer sequence	Reverse primer sequence
CLU	5’-CCAATCAGGGAAGTAAGTACGTC-3’	5’-CTTGCGCTCTTCGTTTGTTTT-3’
MME	5’-AGAAGAAACAGCGATGGACTCC-3’	5’-CATAGAGTGCGATCATTGTCACA-3’
MFGE8	5’-CCTGCCACAACGGTGGTTTAT-3’	5’-CACATTTCGTCTCACAGTGGTT-3’
APOE	5’-GTTGCTGGTCACATTCCTGG-3’	5’-GCAGGTAATCCCAAAAGCGAC-3’
GAL	5’-GGGCAGACTGCTGATCGAG-3’	5’-CCGGTGTCTAAAGGGGATGAT-3’
GAPDH	5’-GGAGCGAGATCCCTCCAAAAT-3’	5’-GGCTGTTGTCATACTTCTCATGG-3’

Cell growth

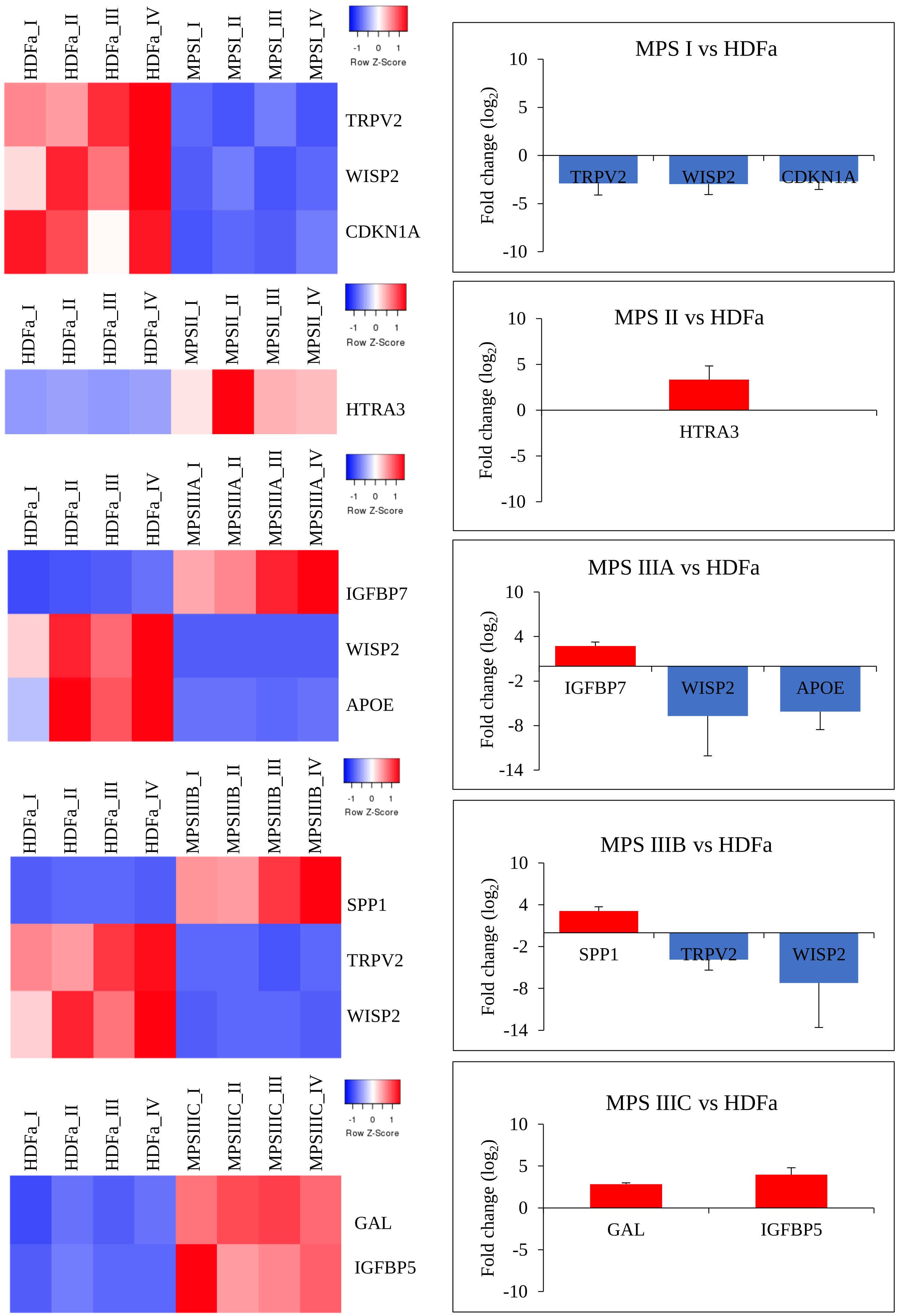
HDFa_I
HDFa_II
HDFa_III
HDFa_IV
MPSI_I
MPSI_II
MPSI_III
MPSI_IV
-1 0 1
Row Z-Score
TRPV2
WISP2
CDKN1A
MPS I vs HDFa
Fold change (log$_2$)
10
5
0
-5
-10
TRPV2
WISP2
CDKN1A
HDFa_I
HDFa_II
HDFa_III
HDFa_IV
MPSII_I
MPSII_II
MPSII_III
MPSII_IV
-1 0 1
Row Z-Score
HTRA3
MPS II vs HDFa
Fold change (log$_2$)
10
5
0
-5
-10
HTRA3
HDFa_I
HDFa_II
HDFa_III
HDFa_IV
MPSIIIA_I
MPSIIIA_II
MPSIIIA_III
MPSIIIA_IV
-1 0 1
Row Z-Score
IGFBP7
WISP2
APOE
MPS IIIA vs HDFa
Fold change (log$_2$)
10
4
-2
-8
-14
IGFBP7
WISP2
APOE
HDFa_I
HDFa_II
HDFa_III
HDFa_IV
MPSIIIB_I
MPSIIIB_II
MPSIIIB_III
MPSIIIB_IV
-1 0 1
Row Z-Score
SPP1
TRPV2
WISP2
MPS IIIB vs HDFa
Fold change (log$_2$)
10
4
-2
-8
-14
SPP1
TRPV2
WISP2
HDFa_I
HDFa_II
HDFa_III
HDFa_IV
MPSIIIC_I
MPSIIIC_II
MPSIIIC_III
MPSIIIC_IV
-1 0 1
Row Z-Score
GAL
IGFBP5
MPS IIIC vs HDFa
Fold change (log$_2$)
10
5
0
-5
-10
GAL
IGFBP5

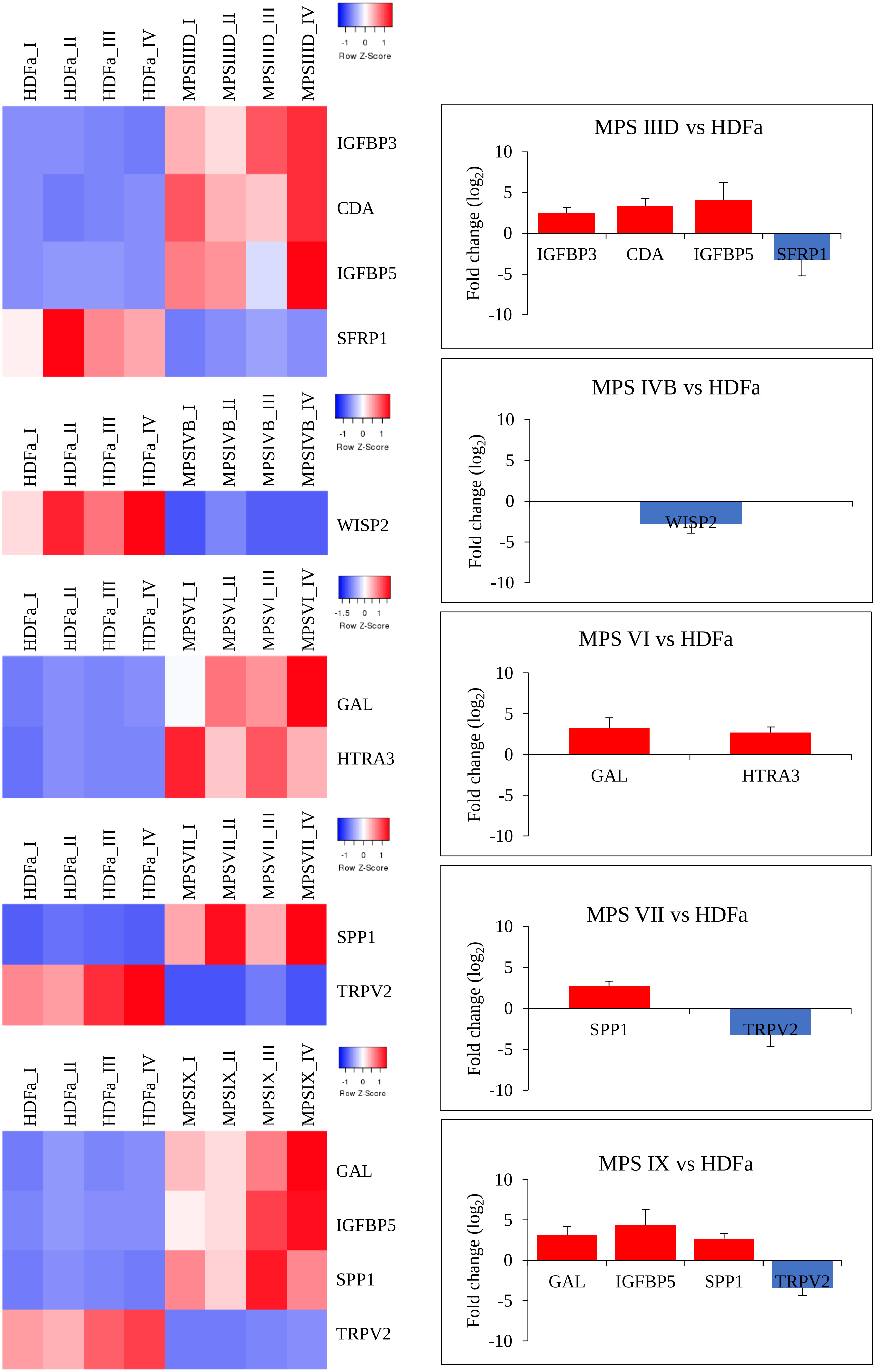
HDFa_I
HDFa_II
HDFa_III
HDFa_IV
MPSIIID_I
MPSIIID_II
MPSIIID_III
MPSIIID_IV
-1 0 1
Row Z-Score
IGFBP3
CDA
IGFBP5
SFRP1
MPS IIID vs HDFa
10
5
0
-5
-10
Fold change (log2)
IGFBP3
CDA
IGFBP5
SFRP1
HDFa_I
HDFa_II
HDFa_III
HDFa_IV
MPSIVB_I
MPSIVB_II
MPSIVB_III
MPSIVB_IV
-1 0 1
Row Z-Score
WISP2
MPS IVB vs HDFa
10
5
0
-5
-10
Fold change (log2)
WISP2
HDFa_I
HDFa_II
HDFa_III
HDFa_IV
MPSVI_I
MPSVI_II
MPSVI_III
MPSVI_IV
-1.5 0 1
Row Z-Score
GAL
HTRA3
MPS VI vs HDFa
10
5
0
-5
-10
Fold change (log2)
GAL
HTRA3
HDFa_I
HDFa_II
HDFa_III
HDFa_IV
MPSVII_I
MPSVII_II
MPSVII_III
MPSVII_IV
-1 0 1
Row Z-Score
SPP1
TRPV2
MPS VII vs HDFa
10
5
0
-5
-10
Fold change (log2)
SPP1
TRPV2
HDFa_I
HDFa_II
HDFa_III
HDFa_IV
MPSIX_I
MPSIX_II
MPSIX_III
MPSIX_IV
-1 0 1
Row Z-Score
GAL
IGFBP5
SPP1
TRPV2
MPS IX vs HDFa
10
5
0
-5
-10
Fold change (log2)
GAL
IGFBP5
SPP1
TRPV2

Cell recognition

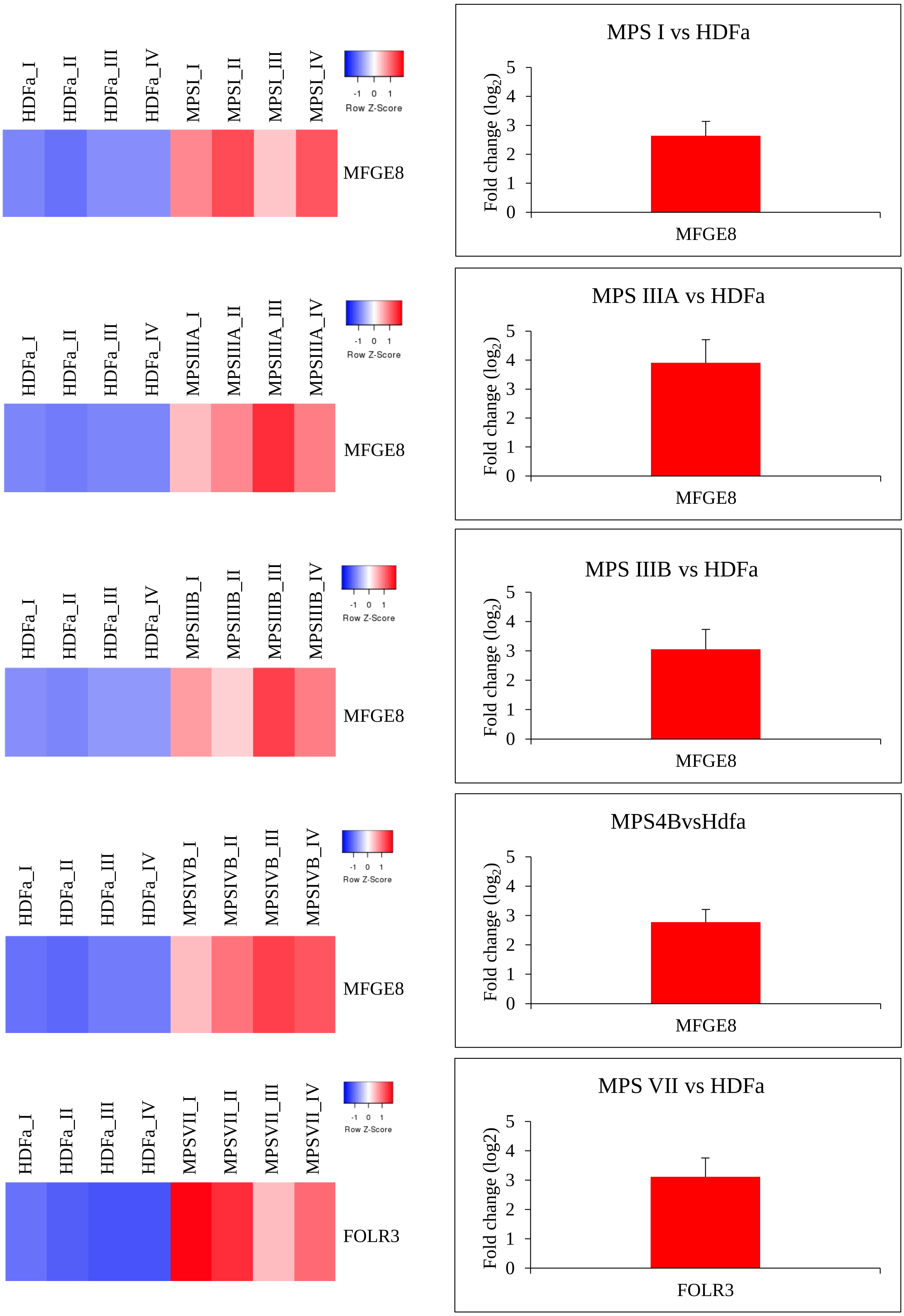

Cell division

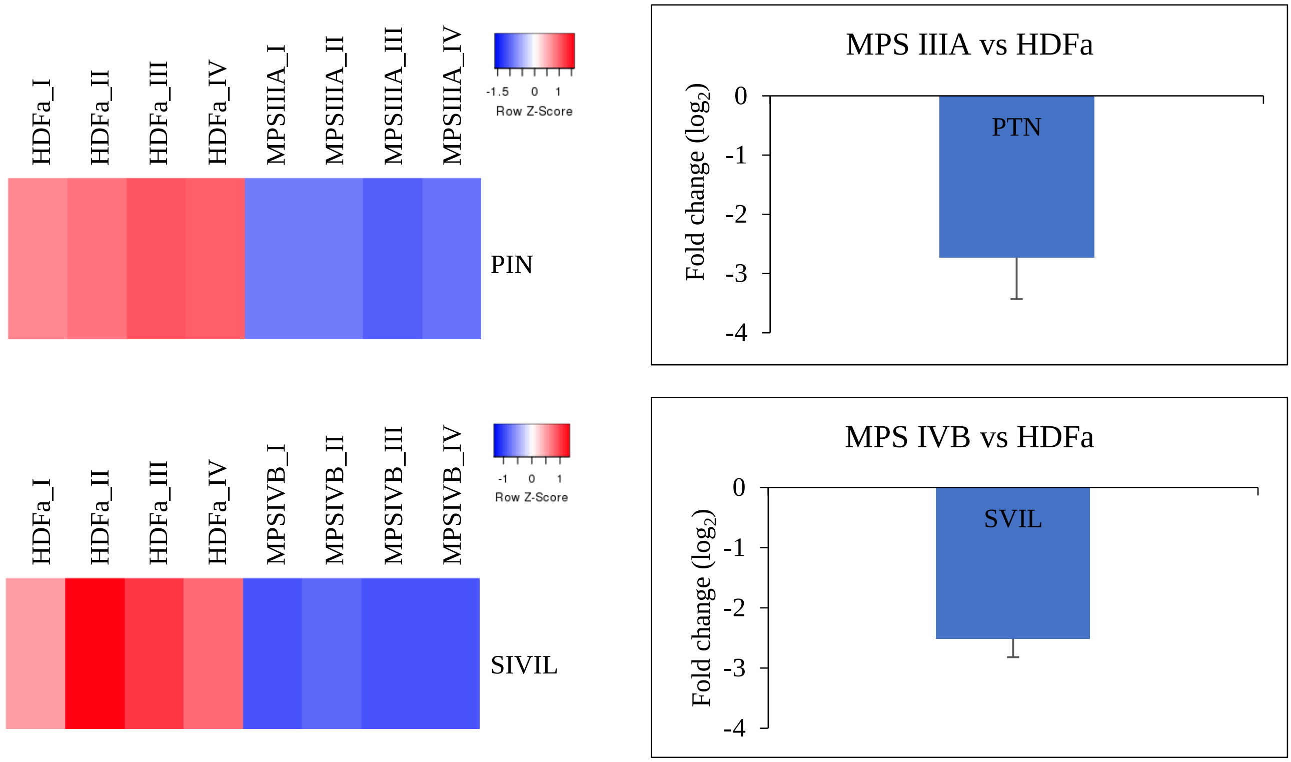

Figure S1: Heat-maps (created with Heatmepper programme) and diagrams presenting genes particularly up- and down-regulated (FDR <0.1; p <0.1; log_2 fold change (FC)>2.5) in each type of MPS compared to HDFa cells taking part in cell growth, recognition and division processes along with an indication of the exact log_2 fold change (FC) value for each gene.
